# Investigation of Electroencephalographic Aspects, Adaptive Features, and Clinical Phenotypes in a Group of Children with Autism—A Pilot Study

**DOI:** 10.3390/clinpract15030050

**Published:** 2025-02-27

**Authors:** Alexandru Capisizu, Leon Zăgrean, Elena Poenaru, Elena Tudorache, Mihaela Anca Bulf, Adriana Sorina Capisizu

**Affiliations:** 1 Dr. Constantin Gorgos Psychiatry Hospital, 030442 Bucharest, Romania; elena.iordan@umfcd.ro (E.T.); ancabulf@gmail.com (M.A.B.); 2 Division of Physiology and Neuroscience, Department of Functional Sciences, Carol Davila University of Medicine and Pharmacy, 050474 Bucharest, Romania; leon.zagrean@umfcd.ro; 3 Discipline of Medical Informatics and Biostatistics, Carol Davila University of Medicine and Pharmacy, 050474 Bucharest, Romania; 4 Department of Radiology and Imagistic Medicine 1, Carol Davila University of Medicine and Pharmacy, 050474 Bucharest, Romania; adriana-sorina.capisizu@drd.umfcd.ro

**Keywords:** autism spectrum disorder, electroencephalography, Adaptive Behavior Assessment System II, neurological examination, children

## Abstract

(1) Background: Autism, as an important global problem that affects many phenotypically different individuals, is associated with electroencephalographic (EEG) abnormalities and adaptability impairment. (2) Materials and Methods: In this retrospective study of a group of 101 autistic children, we aimed to evaluate the presence of EEG abnormalities, adaptive features, and clinical phenotypes via EEG, the Adaptive Behavior Assessment System II (ABAS II) scale, and neurological examination. (3) Results: Our results showed statistically significant associations between the level of adaptability obtained through the ABAS II scale and neurological deficit, specifically in terms of coordination impairment. There were also statistically significant differences between the level of adaptability and clinical phenotypes between autism type groups. (4) Conclusions: This study shows that children with autism are likely to exhibit neurological and adaptive abnormalities. Non-invasive assessment tools, such as EEG recordings, the ABAS II scale, and neurological examination offer valuable support for improved diagnosis and management.

## 1. Introduction

Autism spectrum disorder (ASD) is a neurodevelopmental disorder characterized by social communication impairment and different stereotyped, specific, restricted, repetitive behaviors or interests [1,2], especially in children. According to the Community Report on Autism 2023 from the Centers for Disease Control and Prevention, the prevalence of ASD in children is 2.7% (one in 36 children) worldwide, representing increases of 0.6% and 2.3% compared to 2000 and 2018, respectively [3]. Symptoms of autism have been described as being largely heterogeneous, with various clinical phenotypes associated with the condition [4].

According to the International Classification of Diseases, the tenth revision (ICD-10), autism is classified as a pervasive developmental disorder, which includes several conditions [5]. Of these, childhood autism is associated with deficits in social interaction, communication, as well as restricted behavior, with this developmental pattern presenting before three years of age. For example, atypical autism represents a pervasive developmental disorder that differs from childhood autism, either in terms of age of onset, or by not fulfilling the three above-mentioned sets of diagnostic criteria (impairment in social interaction, communication, and behavior). Moreover, another ICD-10 subcategory, other pervasive developmental disorders, includes individuals who exhibit some autistic elements but do not meet the criteria for a diagnosis of childhood autism or atypical autism.

Autism is an important, complex global problem, with a high prevalence and heterogeneity, affecting a wide spectrum of clinically phenotypically different individuals, and involving impairment in several areas, including electroencephalographic abnormalities and adaptability disorder. Therefore, a variety of non-invasive assessment tools are needed to address autism impairment.

In the scientific literature, studies have shown that electroencephalographic (EEG) abnormalities may be associated with autistic patients, but the prevalence of EEG abnormalities varies greatly (8–80%) [6]. The prevalence of EEG abnormalities is lower in studies that use wake recordings, such as 42% in a study conducted by Romero-Gonzalez et al. [7], than those that use sleep recordings, such as 78% in a study conducted by Santarone et al. [8]. Epileptic-type abnormalities have also been found in EEG recordings, with a prevalence ranging between 28% [8] and 30% [9].

Studies showed that epilepsy was frequently associated with more severe autistic symptoms, a history of regression, lower cognitive abilities or lower intelligence quotient (IQ) levels, poorer adaptive functioning, and less developed language [10,11]. Also, the number of those with autism and epilepsy who presented cognitive impairment was three times higher than those without cognitive impairment [12].

Scientific literature showed a higher incidence of epilepsy and EEG abnormalities in autism [6,7,8,9], both autism and epilepsy have been frequently associated with poorer adaptive functioning [10], and EEG abnormalities alone may indicate poor adaptive functioning in individuals with autism.

The Adaptive Behavior Assessment System II (ABAS II) test has also proven useful in autistic children, owing to the varying degree of impairment of adaptive capacities in such patients [13]. The ABAS II test describes the subject’s normal and abnormal adaptive abilities and behaviors [14], where lower levels of adaptability assessment correlate with a greater degree of impairment in autism [15].

Richard et al. suggested common neurobiological mechanisms linking autism and epilepsy [16]. Núñez-Contreras et al., in a review from 2022 of the main brain mechanisms underlying the association between autism and epilepsy, identified the following pathological causes: abnormalities in the level of various proteins that modulate the first phase of synaptogenesis, mutations in cadherins, protocadherin, and abnormalities in the glutamatergic and GABA systems. Consequently, abnormalities in these systems could produce an imbalance between excitatory and inhibitory networks [17]. The effect of all these abnormal processes, the authors suggested, may lead to a degree of anatomical “over-connectivity” that increases or decreases the efficiency of communication between cortical regions and could represent the basis for the emergence of autism and epilepsy [17]. An imbalance in the excitatory/inhibitory ratio in certain brain areas in patients with autism has also been reported by other authors, such as Kana et al. [18].

Stafstrom et al. explained the decreased seizure threshold and neuronal hyperexcitability that occur in autism, in the case of Fragile X Syndrome, by a disorder of regulation of glutamate-mediated neuronal transmission, and, in the case of Tuberous Sclerosis Complex, by the interruption of the mTOR pathway that leads to the production of cortical tubers, which are epileptogenic neuropathological lesions associated with cognitive deficit and autism [19]. Christensen et al. identified a clear increase in the risk of epilepsy and autism in siblings, concluding that genetic as well as environmental factors may be responsible for the autism–epilepsy association [20].

Scientific literature on children with autism in Romania is limited, especially data on neurological examination and epilepsy in children with autism. In one study by Budișteanu et al. investigating developmental symptoms in a group of 100 children with autism, early clinical signs of autism were identified [21].

To fill this gap in the literature, especially in Romania but not exclusively, there is a need for additional research that uses integrative methods to assess children with autism. Therefore, in this study, we aimed to evaluate the presence of electroencephalographic abnormalities, adaptive features, and clinical phenotypes in a group of children with autism in Romania via EEG, the ABAS II scale, and neurological examination.

This study introduced new findings regarding the associations between ABAS scores and neurological examination results, and the comparisons made between types of autism from an adaptive and phenotypic perspective.

## 2. Materials and Methods

### 2.1. Study Design and Patients

We conducted a retrospective observational study on 101 children diagnosed with autism, who were evaluated by neurological examination and EEG, between February 2021 and April 2023 in the pediatric psychiatry clinic of Psychiatry Hospital “Dr Constantin Gorgos” in Bucharest, Romania. The study was conducted in accordance with the Declaration of Helsinki after receiving approval by the hospital’s ethics committee (Approval number 4747/02.12.2020). The inclusion criteria were patients diagnosed with autism, aged between one year and 18 years, who presented for clinical neurological examination and EEG evaluation, as well as for ABAS II testing, and whose parents or caregivers agreed to take part in the study. Patients previously diagnosed with epilepsy were excluded from the study.

The study included 101 patients, 24 (23.7%) female and 77 (76.3%) male, between the ages of 2 years 2 months and 17 years 11 months. Positive personal history data were recorded, which included any prenatal and perinatal events, such as mother pathology during the gestational period, prenatal suffering of the fetus, intrauterine growth restriction, morphological abnormalities determined by ultrasound or other imaging methods, amniotic fluid abnormalities, premature birth, and perinatal signs, such as cyanosis, unresponsiveness, seizures, or perinatal asphyxia. Family history was considered positive in cases of psychiatric disorders present in family members.

Clinical examination was considered positive for dysmorphic features in cases of facial and body abnormal features, syndromic or non-syndromic, such as stature or weight hypo- or hypertrophies, cranio-facial dysmorphisms (e.g., microcephaly, macrocrania, or craniosynostosis), anomalies of the limbs, or anomalies of the skin (e.g., achromic spots, cafe-au-lait spots, or angiofibromas). Neurological examination was considered positive for abnormalities in the cranial nerves, motility, gait, muscle tone, fine motor skills, coordination, and osteotendinous reflectivity examination.

Data were also collected on whether or not patients received neuroleptic treatment.

### 2.2. Methodology

Patients were evaluated by their attending physician. Data on each patient’s clinical examination and history were retrieved from their medical records, referring to their personal and family history, or obtained from the clinical and neurological examinations. All patients underwent EEG and ABAS II testing.

### 2.3. Medical Investigations

The group of children with autism was evaluated by EEG to identify epileptic and non-epileptic abnormalities, by ABAS II scale to identify adaptive deficits, and by clinical examination to identify clinical phenotypes.

#### 2.3.1. Electroencephalogram

The electroencephalogram consisted of a standard wake EEG, a recording with 19 cephalic bridge-type electrodes arranged in a bipolar montage, using the international 10–20 system, with a reference system and an EKG line, lasting for 15–20 min, in accordance with the International League Against Epilepsy [22]. EEG results were considered positive if the patient showed any abnormality, epileptic or non-epileptic. Epileptic abnormalities were considered spikes, polyspikes, spike and wave complexes, or sharp waves. Non-epileptic abnormalities were considered to be any abnormal focal or generalized activity, producing either a slower or faster type of pathway [23].

#### 2.3.2. Adaptive Behavior Assessment System II

The ABAS II scale describes the subject’s normal and abnormal adaptive skills and behaviors, intellectual disabilities, and adaptive skills, with specificity in differentiating several levels of disability, while representing a robust measurement for a person’s global adaptive functioning [14]. Scores were obtained for individual adaptive skill areas (conceptual, social, and practical) and for the average scale, or the General Adaptive Composite (GAC). Conceptual skills comprised language, reading, writing, and autonomy. Social skills comprised interpersonal relationships, responsibility, and following rules and laws. Practical skills comprised basic activities of daily living and self-care skills.

According to the ABAS II, scaled scores for the adaptive domains and GAC were established as follows: scores of ≥130, 120–129, 110–119, 90–109, 80–89, 71–79, and ≤70 were considered “very high”, “superior”, “above average”, “average”, “below average”, “borderline”, and “extremely low”, respectively [14]. However, for ease of statistical analysis, as most patients presented a borderline or very low score, we regrouped the adaptive and GAC domains as follows: borderline and extremely low scores fell into the “low” category, and the rest of the scaled scores fell into the “above borderline” category.

### 2.4. Statistical Analysis

Statistical data analysis was performed using IBM SPSS version 23 (IBM, Chicago, IL, USA). Qualitative data were reported in terms of frequency and percentage, whereas for quantitative data, normality tests were used in order to determine which tests to report. As such, for quantitative variables, which were normally distributed, mean and standard deviation were reported, whereas for non-normally distributed data, median and interquartile range (IQR: Q1 = 25%, Q3 = 75%) were reported. The chi-squared (X^2^) test was used to assess associations between variables, and the Kruskal–Wallis test was used to assess differences between groups. Differences were considered statistically significant at a *p*-value of <0.05.

## 3. Results

### 3.1. Characteristics of the Patients and Clinical Phenotypes

**Figure 1:**
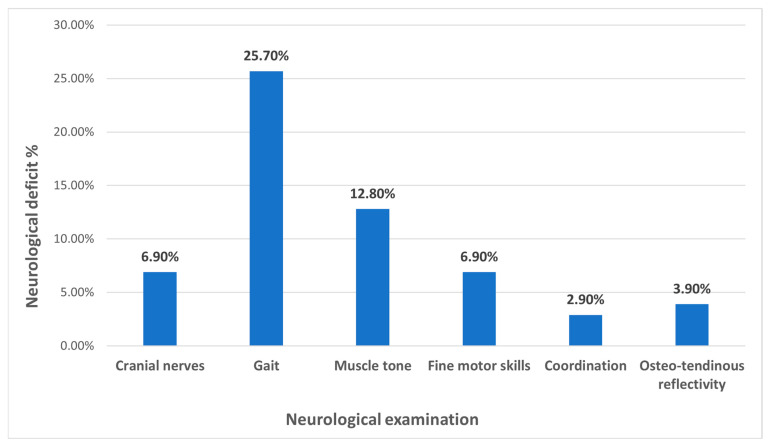
Distribution of patients with neurological examination abnormalities.

Among the patients included in the study, 29 (28.7%) had a positive personal history, and 43 (42.5%) had a positive family history. Positive family history was considered any present or past mental disorder in a close family member, which appeared in childhood or adulthood. During clinical examination, 38 (37.6%) patients presented with dysmorphic features. During neurological examination, seven (6.9%) patients presented with abnormalities of the cranial nerves, 26 (25.7%) with gait disturbance, 13 (12.8%) with muscle tone anomaly, seven (6.9%) with fine motor skills abnormalities, three (2.9%) with coordination disorders, and four (3.9%) with osteotendinous reflectivity abnormalities (Figure 1).

Regarding the primary diagnostic of autism provided by the pediatric psychiatrist, 12 (11.9%) patients were diagnosed with childhood autism, 73 (72.2%) with atypical autism, and 16 (15.9%) with other pervasive developmental disorders. Among all patients, 31 (30.7%) were undergoing neuroleptic treatment.

### 3.2. Electroencephalogram Characteristics

**Figure 2:**
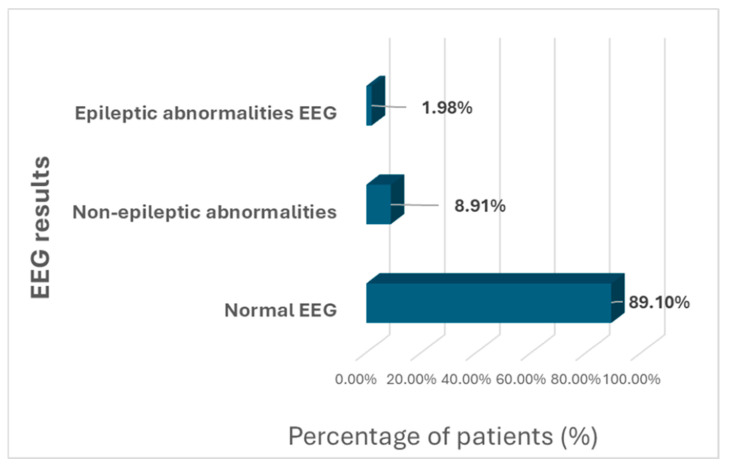
Distribution of patients according to EEG records. EEG: electroencephalography.

Regarding the EEG records, 11 patients (10.89%) showed EEG abnormalities and were divided into non-epileptic abnormalities and epileptic abnormalities. Nine (8.91%) patients presented with EEG non-epileptic abnormalities, of which five (4.95%) presented with focal slow wave bursts, one (0.99%) presented with unilateral focal wave bursts, two (1.98%) presented with generalized wave bursts, and one (0.99%) presented with fast wave bursts. Two (1.98%) patients presented epileptic discharges, which consisted of spike and wave complexes (Figure 2).

Nine (8.91%) patients showed EEG abnormalities in more than one brain area. Of all EEG abnormalities, two (18.1%) were located in the frontal derivations, eight (72.7%) in the central derivations, two (18.1%) in the temporal derivations, four (36.3%) in the parietal derivations, and three (27.2%) were generalized.

There were borderline statistically significant differences for neuroleptic treatment (*p* = 0.087894) between the group with normal EEG (27.8%) and that with abnormalities (54.5%).

Also, there were borderline statistically significant differences for dysmorphic features (*p* = 0.096144) between the group with normal EEG (34.4%) and that with abnormalities (63.6%).

### 3.3. Adaptive Behavior Assessment System II Scale and Clinical Phenotypes

In terms of the patients’ GAC scores, three (2.97%) were average, three (2.97%) were below average, three (2.97%) were borderline, and 92 (91.09%) were extremely low. Based on analysis, there was a statistically significant association between an extremely low GAC score and abnormal coordination (*X*^2^ = 9.98, *p* = 0.01).

Low GAC scores, henceforth referred to as low GAC, showed a statistically significant association with positive family history (*X*^2^ = 4.04, *p* = 0.04), as all 43 (100%) patients with a positive family history had low GAC.

Low GAC also showed a statistically significant association with coordination abnormalities (*X*^2^ = 4.15, *p* = 0.04); of the three patients with coordination abnormalities, two had low GAC.

There was a statistically significant association between Low Conceptual level and the coordination abnormalities (*X*^2^ = 5.29, *p* = 0.02). Of the three patients with coordination abnormalities, two had a Low Conceptual level.

Finally, there was a statistically significant association between Low Social level and the coordination abnormalities (*X*^2^ = 7.01, *p* = 0.008). Also, of the three patients with coordination abnormalities, two had a Low Social level, as seen in Table 1.

### 3.4. Comparing Types of Autism from Adaptive and Clinical Phenotypes Perspectives

A statistically significant difference in age (expressed in months) was found between the atypical autism, childhood autism and other pervasive developmental disorders groups, (*p* = 0.000237). More precisely, the difference was found between childhood autism (164.5 [77.7, 200.0]) and atypical autism (79.0 [50.5, 121.5]), and respectively between atypical autism (79.0 [ 50.5, 121.5]) and other pervasive developmental disorders (152.0 [79.0, 181.2]).

In addition, based on clinical examination, there was a statistically significant difference in positive dysmorphic features between other pervasive developmental disorders, childhood autism and atypical autism groups (*p* = 0.002329). More precisely, the difference was found between other pervasive developmental disorders (31.3%) and childhood autism (83.3%), and between childhood autism (83.3%) and atypical autism (31.5%).

Furthermore, based on neurological examination, statistically significant differences for fine motor skills abnormality were found between other pervasive developmental disorders, childhood autism and atypical autism groups (*p* = 0.005068). More precisely, the difference was found between other pervasive developmental disorders (0%) and childhood autism (33.3%), and between childhood autism (33.3%) and atypical autism (4.1%).

Other statistically significant differences for gait disturbance were found between other pervasive developmental disorders, childhood autism and atypical autism groups (*p* = 0.024908). More precisely, the differences were found between other pervasive developmental disorders (6.3%) and childhood autism (50%).

A statistically significant difference was also registered for the muscle tone anomaly between other pervasive developmental disorders, childhood autism and atypical autism groups (*p* = 0.020671). More precisely, the difference was found between other pervasive developmental disorders (0%) and childhood autism (33.3%), as seen in Table 2.

### 3.5. Differences in ABAS II Score Between Groups

The ABAS II testing results showed statistically significant differences between the childhood autism, atypical autism, and other pervasive developmental disorders groups in terms of GAC score and across all three adaptive domains (conceptual, social, and practical), as seen in Table 3.

There were statistically significant differences for the GAC scores found between other pervasive developmental disorders, childhood autism and atypical autism groups (*p* = 0.013727). More precisely, the differences were found between childhood autism (48.0 [47.2, 48.0]) and other pervasive developmental disorders (59.0 [48.5, 64.0]), respectively, between atypical autism (48.0 [47.0, 55.0]) and other pervasive developmental disorders (59.0 [48.5, 64.0]).

Also, there were statistically significant differences for the conceptual adaptive domain scores found between other pervasive developmental disorders, childhood autism and atypical autism groups, (*p* = 0.005818). More precisely, these differences were found between childhood autism (52.0 [50.2, 52.0]) and other pervasive developmental disorders (60.5 [53.2, 73.0]), respectively, between atypical autism (52.0 [50.0, 56.0]) and other pervasive developmental disorders (60.5 [53.2, 73.0]).

Additionally, there were statistically significant differences for the social adaptive domain scores found between other pervasive developmental disorders, childhood autism and atypical autism groups, (*p* = 0.003943). More precisely, these differences were found between childhood autism (52.0 [52.0, 53.0]) and other pervasive developmental disorders (57.0 [53.2, 76.7]), respectively between atypical autism (53.0 [52.0, 57.0]) and Other pervasive developmental disorders (57.0 [53.2, 76.7]).

Finally, there were statistically significant differences for the practical adaptive domain scores found between other pervasive developmental disorders, childhood autism and atypical autism groups, (*p* = 0.010230). More precisely, these differences were found between childhood autism (49.0 [46.2, 50.0]) and other pervasive developmental disorders (60.5 [50.0, 63.5]), respectively between atypical autism (50.0 [ 47.5, 54.5]) and other pervasive developmental disorders (60.5 [50.0, 63.5]).

## 4. Discussion

Autism, as an important global problem, has seen increases in prevalence and resulting implications in terms of screening and services addressed to such patients [3]. ASD is a neurodevelopmental disorder that affects many phenotypically different individuals, with associated EEG abnormalities and adaptability impairment [4,7]. EEG abnormalities, especially at an early age, can negatively impact brain development, compromising both cognition and behavior. Wake EEG is a non-invasive investigative method that is of great importance in assessing EEG abnormalities in autism. Although the association between autism and EEG abnormalities has been established in many studies [7,8,9,24,25,26], the reported prevalence varies widely between 8% and 80% [26,27].

In the present study, EEG abnormalities were identified in 10.89% of all patients, with 8.9% patients being non-epileptic and 1.9% being epileptic. Hrdlicka reported a similar prevalence of 10.3% [28]. Other studies have found a higher prevalence, such as Romero-Gonzalez et al., who reported a prevalence of 42% of EEG abnormalities in children with autism based on wake EEG results [7]. Santarone et al. reported an even higher prevalence of EEG abnormalities (78%) in a group of preschool autistic children, but they used sleep EEG in their study [8]. Moreover, epileptic-type discharges during sleep were reported in 28.4% of all subjects. Precenzano et al. reported a rate of epileptic discharges of 30% during sleep [9]. Therefore, as highlighted by Capal et al., long-term and sleep EEG records are associated with a greater amount of EEG abnormalities [24].

The current study considered non-epileptic abnormalities consisting of focal and generalized slow or fast wave bursts. Similarly, Santarone et al. reported non-epileptic abnormalities consisting of slow wave bursts in 58% of all subjects, abnormal fast activities in 23%, and asymmetry in 21% [8]. Motor problems, which are as prevalent and functionally impactful as other domains recognized as specifiers to an ASD diagnosis [29], present a significant barrier to activities of daily living, as well as social and cognitive development [30].

In this study, seven (6.9%) patients presented with abnormalities upon cranial nerves examination, 26 (25.7%) with gait disturbance, 13 (12.8%) with muscle tone anomalies, seven (6.9%) with fine motor skills abnormalities, three (2.9%) with coordination disorders, and four (3.9%) with osteotendinous reflectivity anomalies. Motor problems are reported in approximately 1% of autistic individuals [29,31], as well as minor neurological deficits in 73.8% of females and 57.1% of males [32]. De Jong et al. reported cranial nerve dysfunction in 39% of patients, muscle tone anomalies in 87%, fine motor skills abnormalities in 75%, coordination problems in 58%, and abnormal reflexes in 23% [33].

Hughes et al. reported a high percentage of adolescents with ASD with co-occurring neuropsychological conditions [34]. Previous studies that explored the relationship between autism, EEG abnormalities, and adaptive behavior, such as Romero-González et al., found that children with epileptic abnormalities performed worse on adaptive functions assessments [7]. In a study by Lopata et al. [35] that investigated the adaptive functioning of school children under 12 years of age with high-functioning autism, significant deficits on GAC and all three adaptive composites were reported. The results of the present study showed a statistically significant association between abnormal coordination and low GAC, conceptual, and social adaptive levels.

Regarding the significance of autism in relation to EEG, adaptive behavior, and neurological examination brought into perspective by this study, it firstly showed how coordination deficit, revealed through neurological examination, was associated with adaptive impairment. Secondly, results regarding the types of autism and clinical phenotypes highlighted how childhood autism was distinguished from other autistic types, in terms of dysmorphic features, fine motor skills abnormalities, gait disturbance, and muscle tone anomalies. Then, regarding the association between the types of autism and adaptive deficit, the results suggested better adaptive abilities in the other pervasive developmental disorders group. These results are worth considering by other studies that could compare, from an adaptive point of view, different types of autism. Finally, the EEG abnormalities identified presented similar resemblance to results from other studies that used wake EEG.

Despite bringing valuable information to the literature regarding the differences between the types of autism from adaptive and clinical phenotypes perspectives, this study has several limitations. First, the ABAS II scale comprised a subjective evaluation, with the evaluators being the parents, caregivers, or teachers of the subject. In addition, this study used wake EEG, because in our clinical practice, sleep EEG is mainly used in cases where there is a suspicion of epileptic seizures. Finally, this study had a relatively small sample size, which is due to the fact that this was a pilot study. Therefore, future studies should have larger sample sizes, and patients should be evaluated via neurological examination and wake EEG.

## 5. Conclusions

Autism is a complex health problem that must be assessed in terms of phenotypic heterogeneity, EEG abnormalities, and impaired adaptability. By using non-invasive assessment tools of neurological examination and EEG, in addition to the ABAS II scale, this study established statistically significant associations between levels of adaptability, neurological deficits, especially coordination deficit, and clinical phenotypes. Therefore, children with autism should be carefully examined neurologically, and those with particular clinical phenotypes identified, in order to improve diagnosis and case management. Thus, non-invasive assessment tools provide valuable support for understanding clinical characteristics and patient impairments in autism.

## Figures and Tables

**Table 1:** Association between coordination deficit and ABAS II test scores.

Coordination Deficit	ABAS Test	Chi-Squared Test	*p*-Value
	Low GAC	4.15	0.04
	Low Conceptual	5.29	0.02
	Low Social	7.01	0.008

ABAS II: Adaptive Behavior Assessment System II; GAC: General Adaptive Composite.

**Table 2:** Statistical differences between types of autism and clinical phenotypes.

Variable	Childhood Autism (*n* = 12)	Atypical Autism (*n* = 73)	Other Pervasive Developmental Disorders (*n* = 16)	Test	*p*-Value
Age	164.5 [77.7, 200.0]	79.0 [50.5, 121.5]	152.0 [79.0, 181.2]	Kruskal–Wallis	0.000237 Test statistics = 16.696192 (d.f. = 2)
Dysmorphic features	10/12 (83.3%)	23/73 (31.5%)	5/16 (31.3%)	Pearson Chi-Square	0.002329 Test statistics = 12.124357
Fine motor skills abnormality	4/12 (33.3%)	3/73 (4.1%)	0/16 (0%)	Likelihood Ratio	0.005068 Test statistics = 10.569772
Gait disturbance	6/12 (50%)	19/73 (26%)	1/16 (6.3%)	Likelihood Ratio	0.024908 Test statistics = 7.385122
Muscle tone anomaly	4/12 (33.3%)	9/73 (12.3%)	0/16 (0%)	Likelihood Ratio	0.020671 Test statistics = 7.758040

*p*-value of <0.05 is considered statistically significant.

**Table 3:** Statistical comparison of ABAS II test domains between autism types.

ABAS II Test Domain	Kruskal–Wallis Test			*p*-Value
	Childhood Autism (*n* = 12)	Atypical Autism (*n* = 73)	Other Pervasive Developmental Disorders (*n* = 16)	
GAC	48.0 [47.2, 48.0]	48.0 [47.0, 55.0]	59.0 [48.5, 64.0]	0.013727 Test statistics = 8.576710 (d.f. = 2)
Conceptual	52.0 [50.2, 52.0]	52.0 [50.0, 56.0]	60.5 [53.2, 73.0]	0.005818 Test statistics = 10.293582 (d.f. = 2)
Social	52.0 [52.0, 53.0]	53.0 [52.0, 57.0]	57.0 [53.2, 76.7]	0.003943 Test statistics = 11.071848 (d.f. = 2)
Practical	49.0 [46.2, 50.0]	50.0 [47.5, 54.5]	60.5 [50.0, 63.5]	0.010230 Test statistics = 9.164785 (d.f. = 2)

*p*-value of <0.05 is considered statistically significant. GAC: General Adaptive Composite.

## Data Availability

The original contributions presented in this study are included in the article. Further inquiries can be directed to the corresponding authors.

## Abbreviations

The following abbreviations are used in this manuscript:

ABAS II	Adaptive Behavior Assessment System II
ASD	Autism spectrum disorder
EEG	electroencephalography
GAC	General Adaptive Composite

## References

[1] Hirota T., King B.H. (2023). Autism spectrum disorder: A review. JAMA.

[2] Hodges H., Fealko C., Soares N. (2020). Autism spectrum disorder: Definition, epidemiology, causes, and clinical evaluation. Transl. Pediatr..

[3] Maenner M.J., Shaw K.A., Bakian A.V., Bilder D.A., Durkin M.S., Esler A.N., Furnier S.M., Hallas L., Hall-Lande J.A., Hudson A. (2023). Prevalence and characteristics of autism spectrum disorder among children aged 8 years-Autism and Developmental Disabilities Monitoring Network, 11 Sites, United States, 2020. MMWR Surveill. Summ..

[4] Rolland T., Cliquet F., Anney R.J.L., Moreau C.A., Traut N., Mathieu A., Huguet G., Duan J., Warrier V., Portalier S. (2023). Phenotypic effects of genetic variants associated with autism. Nat. Med..

[5] Uysal S. (2019). ICD-10-CM diagnosis coding for neuropsychological assessment. Arch. Clin. Neuropsychol..

[6] Bosetti C., Ferrini L., Ferrari A.R., Bartolini E., Calderoni S. (2024). Children with autism spectrum disorder and abnormalities of clinical EEG: A qualitative review. J. Clin. Med..

[7] Romero-González M., Navas-Sánchez P., Marín-Gámez E., Barbancho-Fernández M.A., Fernández-Sánchez V.E., Lara-Muñoz J.P., Guzmán-Parra J. (2022). EEG abnormalities and clinical phenotypes in pre-school children with autism spectrum disorder. Epilepsy Behav..

[8] Santarone M.E., Zambrano S., Zanotta N., Mani E., Minghetti S., Pozzi M., Villa L., Molteni M., Zucca C. (2023). EEG features in autism spectrum disorder: A retrospective analysis in a cohort of preschool children. Brain Sci..

[9] Precenzano F., Parisi L., Lanzara V., Vetri L., Operto F.F., Pastorino G.M.G., Ruberto M., Messina G., Risoleo M.C., Santoro C. (2020). Electroencephalographic abnormalities in autism spectrum disorder: Characteristics and therapeutic implications. Medicina.

[10] Viscidi E.W., Triche E.W., Pescosolido M.F., McLean R.L., Joseph R.M., Spence S.J., Morrow E.M. (2013). Clinical characteristics of children with autism spectrum disorder and co-occurring epilepsy. PLoS ONE.

[11] Matsuo M., Maeda T., Ishii K., Tajima D., Koga M., Hamasaki Y. (2011). Characterization of childhood-onset complex partial seizures associated with autism spectrum disorder. Epilepsy Behav..

[12] Jokiranta E., Sourander A., Suominen A., Timonen-Soivio L., Brown A.S., Sillanpää M. (2014). Epilepsy among children and adolescents with autism spectrum disorders: A population-based study. J. Autism. Dev. Disord..

[13] Alvares G.A., Bebbington K., Cleary D., Evans K.L., Glasson E.J., Maybery M.T., Pillar S.V., Uljarević M., Varcin K.J., Wray J.A. (2020). The misnomer of ‘high functioning autism’: Intelligence is an imprecise predictor of functional abilities at diagnosis. Autism.

[14] Harrison P.L., Oakland T., București O.S. (2012). ABAS-II: Sistemul de Evaluare a Comportamentului Adaptativ.

[15] Tamm L., Day H.A., Duncan A. (2022). Comparison of adaptive functioning measures in adolescents with autism spectrum disorder without intellectual disability. J. Autism Dev. Disord..

[16] Richard A.E., Scheffer I.E., Wilson S.J. (2017). Features of the broader autism phenotype in people with epilepsy support shared mechanisms between epilepsy and autism spectrum disorder. Neurosci. Biobehav. Rev..

[17] Núñez-Contreras P., Granado-Rocha D., Carvajal-Game M., Torres-Perez A.M. (2022). Aspects of neurodevelopment between autism spectrum disorders and epilepsy. Rev. Mex. Neurocienc..

[18] Kana R.K., Uddin L.Q., Kenet T., Chugani D., Müller R.A. (2014). Brain connectivity in autism. Front. Hum. Neurosci..

[19] Stafstrom C.E., Hagerman P.J., Pessah I.N. (2010). Epilepsy in autism spectrum disorders. Epilepsia.

[20] Christensen J., Overgaard M., Parner E.T., Vestergaard M., Schendel D. (2016). Risk of epilepsy and autism in full and half siblings—A population-based cohort study. Epilepsia.

[21] Budisteanu M., Linca F., Andrei L.E., Mateescu L., Glangher A., Ioana D., Severin E., Riga S., Rad F. (2022). Recognition of early warning signs and symptoms-the first steps on the road to autism spectrum disorder diagnosis. Ann. Ist. Super. Sanita.

[22] Peltola M.E., Leitinger M., Halford J.J., Vinayan K.P., Kobayashi K., Pressler R.M., Mîndruţă I.R., Mayor L.C., Lauronen L.M., Beniczky S. (2023). Routine and sleep EEG: Minimum recording standards of the International Federation of Clinical Neurophysiology and the International League Against Epilepsy. Epilepsia.

[23] Koutroumanidis M., Arzimanoglou A., Caraballo R., Goyal S., Kamińska A.M., Laoprasert P., Oguni H., Rubboli G., Tatum Iv W.O., Thomas P. (2017). The role of EEG in the diagnosis and classification of the epilepsy syndromes: A tool for clinical practice by the ILAE Neurophysiology Task Force (Part 1). Epileptic Disord..

[24] Capal J.K., Carosella C., Corbin E., Horn P.S., Caine R., Manning-Courtney P. (2018). EEG endophenotypes in autism spectrum disorder. Epilepsy Behav..

[25] Mulligan C.K., Trauner D.A. (2014). Incidence and behavioral correlates of epileptiform abnormalities in autism spectrum disorders. J. Autism Dev. Disord..

[26] Baird G., Robinson R.O., Boyd S., Charman T. (2006). Sleep electroencephalograms in young children with autism with and without regression. Dev. Med. Child. Neurol..

[27] Boutros N.N., Lajiness-O’Neill R., Zillgitt A., Richard A.E., Bowyer S.M. (2015). EEG changes associated with autistic spectrum disorders. Neuropsychiatr. Electrophysiol..

[28] Hrdlicka M. (2008). EEG abnormalities, epilepsy and regression in autism: A review. Neuroendocrinol. Lett..

[29] Licari M.K., Alvares G.A., Varcin K., Evans K.L., Cleary D.B., Reid S.L., Glasson E.J., Bebbington K., Reynolds J.E., Wray J.A. (2020). Prevalence of motor difficulties in autism spectrum disorder: Analysis of a population-based cohort. Autism Res..

[30] Miller H.L., Licari M.K., Bhat A., Aziz-Zadeh L.S., van Damme T., Fears N.E., Cermak S.A., Tamplain P.M. (2024). Motor problems in autism: Co-occurrence or feature?. Dev. Med. Child Neurol..

[31] Miller H.L., Sherrod G.M., Mauk J.E., Fears N.E., Hynan L.S., Tamplain P.M. (2021). Shared features or co-occurrence? Evaluating symptoms of developmental coordination disorder in children and adolescents with autism spectrum disorder. J. Autism Dev. Disord..

[32] Ben-Itzchak E., Ben-Shachar S., Zachor D.A. (2013). Specific neurological phenotypes in autism spectrum disorders are associated with sex representation. Autism Res..

[33] De Jong M., Punt M., De Groot E., Minderaa R.B., Hadders-Algra M. (2011). Minor neurological dysfunction in children with autism spectrum disorder. Dev. Med. Child Neurol..

[34] Hughes M.M., Shaw K.A., Patrick M.E., DiRienzo M., Bakian A.V., Bilder D.A., Durkin M.S., Hudson A., Spivey M.H., DaWalt L.S. (2023). Adolescents with autism spectrum disorder: Diagnostic patterns, co-occurring conditions, and transition planning. J. Adolesc. Health.

[35] Lopata C., Fox J.D., Thomeer M.L., Smith R.A., Volker M.A., Kessel C.M., McDonald C.A., Lee G.K. (2012). ABAS-II ratings and correlates of adaptive behavior in children with HFASDs. J. Dev. Phys. Disabil..

